# Myocardial Injury at Early Stage and Its Association With the Risk of Death in COVID-19 Patients: A Hospital-Based Retrospective Cohort Study

**DOI:** 10.3389/fcvm.2020.590688

**Published:** 2020-10-30

**Authors:** Lin Fu, Xiu-Yong Li, Jun Fei, Ying Xiang, Hui-Xian Xiang, Meng-Die Li, Fang-Fang Liu, Ying Li, Hui Zhao, De-Xiang Xu

**Affiliations:** ^1^Second Affiliated Hospital, Anhui Medical University, Hefei, China; ^2^School of Public Health, Anhui Medical University, Hefei, China; ^3^The Second People's Hospital of Fuyang City, Fuyang, China; ^4^Union Hospital, Huazhong University of Science and Technology, Wuhan, China

**Keywords:** severe acute respiratory syndrome coronavirus-2 (SARS-CoV-2), coronavirus disease 2019 (COVID-19), myocardial injury, prognosis, death

## Abstract

**Background:** There are growing evidence demonstrating that coronavirus disease 2019 (COVID-19) is companied by acute myocardial injury. However, the associations of SARS-CoV-2-induced myocardial injury with the risk of death and prognosis after discharge in COVID-19 patients are unclear.

**Methods:** This prospective cohort study analyzed 355 COVID-19 patients from two hospitals in different regions. Clinical and demographic information were collected and prognosis was followed up.

**Results:** Of 355 hospitalized patients with COVID-19, 213 were mild, 90 severe, and 52 critically ill patients. On admission, 59 (16.7%) patients were with myocardial injury. Myocardial injury was more popular in critically ill patients. Univariate and multivariate logistic regression revealed that male, older age and comorbidity with hypertension were three crucial independent risk factors predicting myocardial injury of COVID-19 patients. Among 59 COVID-19 patients with myocardial injury, 25 (42.4%) died on average 10.9 days after hospitalization. Mortality was increased among COVID-19 patients with myocardial injury (42.4 vs. 3.38%, *RR* = 12.542, *P* < 0.001). Follow-up study observed that 4.67% COVID-19 patients with myocardial injury were not fully recovered in 14 days after discharge.

**Conclusion:** Myocardial injury at early stage elevates mortality of COVID-19 patients. Male elderly patients with hypertension are more vulnerable to myocardial injury. SARS-CoV-2-induced myocardial injury has not completely recovered in 14 days after discharge.

## Introduction

Coronavirus Disease 2019 (COVID-19) is a newly recognized infectious disease caused by the newly discovered Severe Acute Respiratory Syndrome- Coronavirus-2 (SARS-CoV-2). COVID-19 firstly broke out in Wuhan city, Hubei province in China since December 2019 (1). Now, it has been pandemic all over the world. Up to April 19th, China has cumulatively diagnosed 84,225 cases and over 4,642 deaths, while the number of cases in other countries is growing rapidly with a total of 2,277,363 confirmed cases and 157,572 death cases (2). American and Europe have gradually become the epicenter of the pandemic and the entire world is suffering great public health crisis from SARS-CoV-2, which is more severe than 2003 SARS disaster (3).

Some studies have found that SARS-CoV-2 transmitted through not only droplets or direct contact but also feces (4, 5). Evidence pointing to the person-to-person transmission has occurred among close contacts in hospital and family (6). As reported by other researchers and our team found that patients with COVID-19 present primarily with fever, diarrhea, fatigue, dry cough, lymphopenia and radiographic evidence of pneumonia (7–9). The Chinese Center for Disease Control and Prevention recently revealed that the risk of death of mild COVID-19 patients was relatively low in a large Chinese population-based study, the fatality rate was significantly elevated among critically ill COVID-19 cases (10). Previous studies found that SARS-CoV-2 mainly evoked severe acute respiratory syndrome. More and more researches revealed that most cases were prone to suffer multiple organ injuries, such as immune system disorder, acute kidney injure and even liver dysfunction (11–13). The clinical characteristics of myocardial injury caused by SARS-CoV-2 are gradually being recognized. However, the associations between SARS-CoV-2-induced myocardial injury with the risk of death and the prognosis after discharge in patients with COVID-19 remain unknown.

The aim of this study was to investigate SARS-CoV-2-induced myocardial injury, its associations with the risk of death and prognosis in the short term after discharge. Our results suggested that male elderly COVID-19 patients with hypertension were more vulnerable to myocardial injury. We found that myocardial injury at early stage increased the risk of death among COVID-19 patients. This study firstly provides evidence that SARS-CoV-2 induced myocardial injury has not completely recovered in 14 days after discharge.

## Methods

### Study Design and Participants

In the present study, 200 patients with COVID-19 were recruited in Union Hospital of Huazhong University of Science and Technology in Wuhan city from January 1 to January 30, 2020. Another 155 patients with COVID-19 were recruited from the Second People's Hospital of Fuyang City in Anhui province. Union Hospital of Huazhong University of Science and Technology and Second People's Hospital of Fuyang City were assigned responsibility for the treatment of patients with COVID-19 by the Anhui government and Wuhan government. All patients were laboratory confirmed positive of SARS-CoV-2 injection by RT-PCR on pharyngeal swab specimens. Diagnosis and treatment of COVID-19 were based on the New Coronavirus Pneumonia Prevention and Control Program (6th edition) published by the National Health Commission of China. Laboratory examination, epidemiological investigation, imaging examination, electrocardiogram assessment and etiological examination were performed in all COVID-19 patients. When patients were diagnosed with SARS-CoV-2 injection, patients were isolated and began to cure based on the guide. Next, conventional observation of patients' conditions, antivirotic treatment, antibacterial treatment when patients accompanied with bacterial injection were carried among COVID-19 patients, such as interferon, lopinavir, ritonavir, and chloroquine phosphate were used. Moreover, some severe and critically ill patients may receive respiratory support with mechanical ventilation, rescue treatment using ECMO, circulation supportive or Chinese medicine treatment. Except for the above-mentioned drugs, glucocorticoid was also used in the severe and critically ill patients. The patients could be discharged who met the following criteria: body temperature was normal more than 3 days, respiratory symptoms were ameliorated, diffuse infiltration was absorbed using CT imaging and two consecutive nucleic acid detections of SARS-CoV-2 virus in nasopharyngeal swab and sputum were negative with 24 h interval. There was no death case with COVID-19 in the Second People's Hospital of Fuyang City. At last, 150 cured cases were performed follow-up examination in 14 days after discharge in the Second People's Hospital of Fuyang City, biochemical indexes and blood routine were detected. This study was approved by the institutional ethics board of Union Hospital of Huazhong University of Science and Technology, and Second People's Hospital of Fuyang City. All COVID-19 patients were eligible in this study. Oral consent was obtained from patients or patients' next of kin.

### Data Collection

The medical record of each COVID-19 patient was collected. Patient's data including demographics, comorbidities (chronic obstructive pulmonary disease, hepatic disease, cardiovascular disease, hypertension, diabetes and other disease), patient's signs and symptoms, and laboratory test results were collected. The date of onset and outcomes were recorded.

### Laboratory Testing

Patient's pharyngeal swab specimens were measured. Real-time RT-PCR was used to detect viral nucleic acid using COVID-19 nucleic acid detection kits following experimental instructions (Shanghai bio-germ Medical Technology Co Ltd). Troponin (Tn), aspartate aminotransferase (AST), creatine kinase (CK), creatine kinase isoenzyme (CKMB), lactate dehydrogenase (LDH), oxygenation index (PaO_2_/FiO_2_), C-reactive protein (CRP) were examined on admission. Myocardial injury was defined as troponin beyond normal range (14). A complete blood routine and blood chemistries were conducted in all COVID-19 patients.

### Statistical Analysis

All statistical analyses were performed using SPSS19.0 software. Categorical variables were expressed with frequencies and percentages. Continuous variables were shown using median and mean values. All categorical variables were compared for the study outcome by the Fisher exact test or χ^2^ test, and continuous variables were compared using the *t*-test or the Mann-Whitney *U* test, as appropriate. Logistic regression analysis between myocardial injure with different parameters and the prognosis were performed. Statistical significance was determined at *P* < 0.05.

## Results

### Demographic and Clinical Characteristics

All 355 COVID-19 patients' clinical information was collected and evaluated. As shown in Table 1, mild case, defined as oxygenation index higher than 300, was 213 (60.0%). For severe case, whose oxygenation index was from 200 to 300, was 90 (25.4%). For critically ill case, whose oxygenation index was lower than 200, accounted for 14.6% (Table 1). Moreover, the demographic characteristics were then analyzed. As shown in Table 2, 162 (45.6%) were female and 193 (54.4%) were male. There were 96 patients younger than 39 years old, 144 patients aged between 40 and 59, and 115 patients older than 60 years old. Of 355 patients with COVID-19, 230 (64.8%) patients had hypertension, 208 (58.6%) patients had diabetes, and 20 (5.63%) had chronic heart disease.

**Table 1:** The associations between the severity and myocardial injury indexes.

**Parameters**	**Mild**	**Severe**	**Critically ill**
Cases, *N*	213	90	52
CK (U/L)	70.0 (47.0, 101.0)	72.0 (47.3, 160.0)	149 (70.0, 319.0)^**^^##^
CKMB, *N* (%)	69 (33.7)	51 (59.3)	38 (74.5)^*^
LDH (U/L)	222.0 (186.0, 272.0)	299.0 (226.0, 370.0)^**^	442.0 (277.5, 630.3)^**^^##^
AST (U/L)	26.0 (20.0, 35.0)	29.0 (22.8, 54.0)^*^	49.0 (35.0, 80.0)^**^^##^
Tn (ng/mL)	0.01 (0, 0.02)	0.04 (0.02, 0.06)^*^	0.07 (0.05, 0.12)^**^^##^
Myocardial injury cases (%)	22 (10.3)	19 (21.1)^*^	18 (34.6)^**^^#^

*Compared with “Mild”*,

* *P < 0.05*,

** P < 0.01;

*Compared with “Severe”*,

# *P < 0.05*,

## *P < 0.01*.

*Myocardial injury was defined as troponin beyond normal range*.

**Table 2:** The effects of demographic characteristics and complications on myocardial injury indexes.

	**Cases**	**CK (U/L)**	**CKMB, *N* (%)**	**LDH (U/L)**	**AST (U/L)**	**Tn (ng/mL)**
**Gender**						
Female	162	59.5 (40.0, 97.0)	79 (49.1)	236.0 (188.5, 341.0)	26.0 (20.0, 39.0)	0.04 (0.02, 0.06)
Male	193	92.0 (59.5, 153.5)^**^	76 (41.5)	254.0 (201.5, 337.5)	30.0 (23.0, 47.0)^*^	0.05 (0.02, 0.07)
**Age**						
<39	96	75.5 (41.5, 101.5)	19 (19.8)	209.0 (172.0, 261.0)	23.0 (19.0, 29.0)	0.03 (0.01, 0.05)
40–59	144	69.0 (47.0, 114.0)^**^	54 (37.8)^**^	246.0 (200.3, 327.5)^**^	28.0 (22.0, 45.0)^*^	0.05 (0.02, 0.08)^**^
>60	115	99.0 (58.0, 194.0)^**^^##^	82 (71.3)^**^^##^	296.0 (223.0, 450.5)^**^^##^	36.0 (23.0, 57.5)^**^^##^	0.08 (0.06, 0.10)^**^^##^
**Hypertension**						
Yes	230	72.0 (47.0, 166.0)	89 (73.0)	270.0 (189.0, 403.0)	31.5 (21.0, 58.8)	0.05 (0.02, 0.07)
No	125	77.0 (50.0, 122.0)	69 (30.7)^**^	239.0 (195.8, 312.8)	27.0 (21.0, 39.0)^*^	0.04 (0.01, 0.06)
**Diabetes**						
Yes	208	89.0 (50.0, 170.0)	95 (66.4)	270.0 (177.3, 389.5)	32.0 (23.0, 58.0)	0.04 (0.02, 0.07)
No	147	72.0 (49.0, 113.8)^*^	63 (30.9)	237.0 (197.0, 306.0)	26.0 (21.0, 37.5)^**^	0.05 (0.02, 0.08)
**Heart disease**						
Yes	20	78.0 (47.0, 184.0)	12 (63.2)	346.0 (223.0, 480.0)	35.0 (21.0, 71.0)	0.06 (0.03, 0.09)
No	335	77.0 (49.0, 125.5)	146 (44.5)	245.0 (193.8, 323.8)^*^	28.0 (21.0, 43.0)	0.04 (0.01, 0.07)^*^

*Cases in gender, compared with “Female”*,

* *P < 0.05*,

** *P < 0.01*.

*Cases in age, compared with “<39”*,

* *P < 0.05*,

** *P < 0.01; compared with “40–59”*,

## *P < 0.01*.

*Cases in hypertension, diabetes, and heart disease, compared with “Yes”*,

* *P < 0.05*,

** *P < 0.01*.

### Association of Myocardial Injury With the Severity of COVID-19 Patients

COVID-19 firstly broke out in Wuhan City of China. Because the number of patients was large and medical resources were limited, only electrocardiogram assessment of partially patients were performed (data not shown). We found that the patients with left and right ventricular dysfunction were more in severe and critically ill patients than those in mild patients with COVID-19. Diastolic dysfunction was observed in only a small number of patients with COVID-19. Only a few patients had mild pericardial effusion without other clinical or electrocardiographic signs of pericarditis. The association between myocardial injury and the severity of COVID-19 was evaluated in patients. Myocardial injury indexes, including Tn, CK, CKMB, LDH, and AST, were analyzed. As shown in Table 1, the level of CK were higher in critically ill patients than those in mild and severe patients. The number of CKMB-positive patients were more in critically ill patients than those of mild patients. The levels of LDH and AST were the lowest in the mild patients with COVID-19. Moreover, the levels of LDH and AST were higher in the critically ill patients than those of in severe patients. The levels of Tn were gradually increased in parallel with the severity of COVID-19. The results indicated that 22 (10.3%) COVID-19 patients were with myocardial injury at early stage in mild patients. Nineteen (21.1%) patients with myocardial injury were in severe patients and 18 (34.6%) patients with myocardial injury were in critically ill patients. Furthermore, the associations between oxygenation index and myocardial injury markers were analyzed. As shown in Supplementary Table 1, no association between oxygenation index with CK and CKMB was observed. There was a negative association between AST (*r* = −0.249, *P* = 0.001), LDH (*r* = −0.431, *P* < 0.001) and Tn (*r* = −0.221, *P* = 0.038) with oxygenation index among COVID-19 patients. Additionally, the associations between inflammatory cytokine and myocardial injury markers were analyzed. The results indicated that CRP was positively correlated with AST (*r* = 0.241, *P* = 0.004), LDH (*r* = 0.457, *P* < 0.001) and CK (*r* = −0.198, *P* = 0.018) (Supplementary Table 1).

### Male Elderly COVID-19 Patients With Hypertension Are More Vulnerable to Myocardial Injury

The effects of demographic characteristics on myocardial injury markers were analyzed. As shown in Table 2, the level of CK were higher in males than in females. There was no difference of CKMB, LDH, AST, and Tn between females and males. Further analysis showed that CK, CKMB, LDH, AST, and Tn were lower in patients younger than 39 years old than those of older patients. Moreover, we found that CK, CKMB, LDH, AST, and Tn were higher in patients older than 60 than those between 40 and 59 years old (Table 2). The effects of comorbidities on myocardial functional indexes were then analyzed. As shown in Table 2, CKMB-positive patients were more in COVID-19 patients with hypertension than those without hypertension. Besides, the level of CK was elevated in COVID-19 patients with diabetes compared with those without diabetes. LDH and AST were increased in COVID-19 patients with chronic heart disease compared with those without heart disease. There was no difference of Tn in patients with hypertension and diabetes or not. The levels of Tn were higher in patients with heart disease than those in patients without heart disease. In addition, the risk factors of myocardial injury were analyzed using univariate and multivariate logistic regression among COVID-19 patients. In the univariate logistic regression analysis, the *OR* of male gender was 2.012 (95% CI: 1.125, 3.599), the *OR* of age was 1.434 (95% CI: 1.041, 1.976) and the *OR* of hypertension was 3.393 (95% CI: 1.441, 7.989) for myocardial injury (Table 3). In the multivariate logistic regression analysis, the *OR* of male gender was 2.349 (95% CI: 1.135, 4.312), the *OR* of age was 1.332 (95% CI: 1.014, 2.123) and the *OR* of hypertension was 2.958 (95% CI: 1.331, 5.636) for myocardial injury (Table 3).

**Table 3:** Logistic regression analysis the risk factors of myocardial injury among COVID-19 patients.

	**Univariate logistic regression analysis**				**Multivariate logistic regression analysis**			
	**β**	**Wald**	***P-value***	**OR (95% CI)**	**β**	**Wald**	***P-value***	**OR (95% CI)**
Male	0.699	5.556	0.018	2.012 (1.125, 3.599)	0.717	5.662	0.017	2.349 (1.135, 4.312)
Age	−1.304	5.003	0.028	1.434 (1.041, 1.976)	0.031	12.136	0.001	1.332 (1.014, 2.123)
Hypertension	1.222	7.814	0.005	3.393 (1.441, 7.989)	1.160	6.773	0.009	2.958 (1.331, 5.636)
Diabetes	0.660	3.118	0.077	1.936 (0.930, 4.029)	0.498	1.658	0.198	1.646 (0.771, 3.513)
Heart disease	−0.016	0.000	0.984	0.984 (0.202, 4.788)	−0.791	1.083	0.298	0.454 (0.102, 2.010)

### Myocardial Injury at Early Stage Elevates the Risk of Death of COVID-19 Patients

The effects of myocardial injury at the early stage on the risk of death were analyzed using multivariate logistic regression after adjusted age, sex, and comorbidities. An shown in Table 4, among 59 COVID-19 patients with myocardial injury, 42.4% were died. In multivariate logistic regression analysis, the fatality rate was higher among COVID-19 patients with myocardial injury than those without myocardial injury (42.4 vs. 3.38%; *RR* = 12.542, *95% Cl*: 6.367, 24.708; *P* < 0.001).

**Table 4:** The association between myocardial injury and death risk among COVID-19 patients.

**Myocardial injury**	**Cases**	**Death (%)**	**RR (95% CI)**	***P-value***
Yes	59	25 (42.4)	12.542 (6.367, 24.708)	<0.001
No	296	10 (3.38)	1	—

*Adjusted for age, sex, and comorbidities*.

### Myocardial Markers Remain Abnormal in 14 Days After Discharge

The recovery of myocardial injury was investigated in every patient with COVID-19. The levels of myocardial markers were compared between on admission and in 14 days after discharge in the Second People's Hospital of Fuyang City. As shown in Table 5, there was no significant difference in the levels of CK, CKMB, AST, and Tn among COVID-19 patients between on admission and after discharge, whereas LDH was decreased in 14 days after discharge than on admission. On admission, 5 (3.3%) cases with CK, 9 (6.1%) cases with CKMB, 56 (38.1%) cases with LDH, 19 (12.3%) cases with AST, and 20 (13.0%) cases with Tn were above the normal range. In all, there was 20 (13.0%) COVID-19 patients with myocardial injury. The prognosis of COVID-19 patients' myocardial injury markers was followed up in 14 days after discharge in the Second People's Hospital of Fuyang City. We found that 2 (1.33%) patients with CKMB, 25 (16.7%) patients with LDH, 25 (15.3%) patients with AST, and 7 (4.67%) patients with Tn remained above the normal range. Further analysis showed that 4.67% patients with COVID-19 continuously accompanied with myocardial injury in 14 days after discharge (Table 5).

**Table 5:** Myocardial injury indexes on admission and after discharge among COVID-19 patients.

**Myocardial injury indexes**	**On admission (*****N*** **=** **154)**			**Discharge (*****N*** **=** **150)**		
	**Median**	**Below the range, *N* (%)**	**Above the range, *N* (%)**	**Median**	**Below the range, *N* (%)**	**Above the range, *N* (%)**
CK (U/L)	65.0 (44.0, 96.0)	45 (29.8)	5 (3.3)	63.0 (47.0, 82.0)	43 (28.6)	0^#^
CKMB (U/L)	8.0 (5.0, 13.0)	0	9 (6.1)	7.0 (4.0, 11.0)	0	2 (1.33)^#^
LDH (U/L)	230.0 (194.0, 278.0)	1 (0.7)	56 (38.1)	203.0 (176.0, 242.0)^**^	0	25 (16.7)^##^
AST (U/L)	24.0 (20.0, 31.0)	23 (15.0)	19 (12.3)	22.0 (19.0, 30.0)	11 (7.5)	23 (15.3)
Tn (ng/mL)	0.04 (0.02, 0.06)	0	20 (13.0)	0.03 (0.01, 0.04)	0	7 (4.67)^#^

*Compared with “Median values” among COVID-19 patients on admission*,

** *P < 0.01*.

*Compared with “Above the range” among COVID-19 patients on admission*,

# *P < 0.05*,

## *P < 0.01*.

## Discussion

This study mainly investigated SARS-CoV-2-induced myocardial injury, its associations with mortality and the prognosis in 14 days after discharge. The major results of this study include: (1) Myocardial injury is more popular in the critically ill patients with COVID-19; (2) Male elderly COVID-19 patients with hypertension are more vulnerable to myocardial injury; (3) Myocardial injury at early stage elevates the risk of death of COVID-19 patients; (4) Myocardial injury of 4.67% COVID-19 patients has not completely recovered in 14 days after discharge.

More and more studies have demonstrated that COVID-19 patients were accompanied with multiple organ injuries, mainly including acute liver injury, acute kidney injury, respiratory failure and even lymphopenia (12, 13, 15). In the present study, myocardial injury was evaluated through measuring biochemical indexes, such as CK, CKMB, LDH, AST, and Tn. We found that these five myocardial injury markers were higher in critically ill patients than those in mild and severe patients with COVID-19. Moreover, the cases of myocardial injury were more in critically ill patients than those in mild and severe patients with COVID-19. Myocardial injury was more popular in critically ill patients. These results provide evidence that myocardial injury at early stage is positively associated with the severity of COVID-19 patients.

The previous studies have revealed that older age patients have more severe symptoms and signs (12, 16). In the present study, the effects of demographic characteristics on myocardial injury were analyzed. Although no difference of CKMB, LDH, and Tn were observed between females and males, CK and AST was higher in males than those in females. In addition, the number of CKMB-positive cases was more in older patients than younger patients. Several reports indicated that comorbidities elevated the risk of death and the severity of COVID-19 patients (17, 18). The present study found that 64.8% patients were with hypertension, 58.6% patients were with diabetes and 5.63% patients were with chronic heart disease. In order to investigate the influence of comorbidities on myocardial injury, the markers of myocardial injury were analyzed among COVID-19 patients. This study found that the number of CKMB-positive cases was more in patients with hypertension than those without hypertension. In addition, the level of serum CK was slightly increased in patients with diabetes. The level of serum LDH was significantly higher in patients with chronic heart disease as compared with those without heart disease. The level of Tn was obviously increased in patients with heart disease than those without heart disease. These results indicate that male, older age and comorbidities may aggravate myocardial injury of COVID-19 patients. In order to further analyze the associations between myocardial injury with demographic characteristics and comorbidities, the univariate and multivariate logistic regression were performed. Our results indicated that male, older age, comorbidity with hypertension were three independent risk factors of myocardial injury. Generally speaking, male elderly COVID-19 patients with hypertension are more vulnerable to myocardial injury.

The effect of myocardial injury on the prognosis of COVID-19 patients is not yet clear. The association between myocardial injury and the risk of death was analyzed among COVID-19 patients. The present study found that the mortality was higher in COVID-19 patients with myocardial injury than those without myocardial injury. Myocardial injury on admission obviously elevates the risk of death among COVID-19 patients. This is an urgent issue which is worthy of studying whether myocardial injury recovers during a short-period after discharge. In this work, 150 COVID-19 patients were tracked and markers of myocardial injury were detected. The rate of myocardial injury between on admission and in 14 days after discharge were compared among COVID-19 patients from the Second People's Hospital of Fuyang City. Although no remarkably difference of the levels of serum CK, CKMB, AST, and Tn were observed between on admission and in 14 days after discharge, the level of serum LDH was obviously decreased in 14 days after discharge. In spite of the abnormal number of CKMB, LDH and Tn were decreased, 1.33% patients with CKMB, 16.7% patients with LDH, and 4.67% patients with Tn were above the normal range. Our results indicate that myocardial injury of 4.67% patients with COVID-19 were not fully recovered in 14 days after discharge. Therefore, whether SARS-CoV-2 causes continuous myocardial injury is needed to perform further follow-up research in the future clinical work.

The mechanism of which SARS-CoV-2 induces myocardial injury is scarcely clear. The previous study found that CRP was evidently increased in the critically ill patients (19, 20). CRP is an acute-phase protein in response to inflammatory cytokines after infections. High level of CRP partially reflects the severity of inflammation and evokes cytokine storm, which largely enhances vascular permeability and impairs organ function. Our results found that CRP was positively correlated with the levels of AST, LDH and CK, indicating that SARS-CoV-2-induced cytokine storm may be one of the mechanisms of myocardial injury. Both our research and other team found that SARS-CoV-2 injection reduced oxygenation index and caused respiratory function failure (9). Continuous blood hypoxia induces acidosis and excess generation of reactive oxygen species, ultimately damaging myocardial cell. In the present study, we found that oxygenation index was negatively associated with AST, LDH and Tn among COVID-19 patients, suggesting that respiratory function failure may contribute, at least partially, to SARS-CoV-2-evoked myocardial injury. Increasing data demonstrate that angiotension converting enzyme (ACE)2, as a receptor for SARS-CoV-2, exerts a significant role in the pathogenesis of COVID-19 patients (21–23). Recently, a report found that ACE2 was also expressed in cardiocytes (24). Hence, these evidences don't exclude that SARS-CoV-2 evokes myocardial injury partially through directly damaging myocardial cells.

In brief, this research mainly analyzed the associations between SARS-CoV-2-induced myocardial injury with mortality and the prognosis in 14 days after discharge based on a retrospective cohort study. Nevertheless, there are some flaws in this study. Firstly, the patients were only from two different regions and the sample size was mild. A larger sample size from multicenter in China is needed in the future study. Secondly, COVID-19 firstly broke out in Wuhan City of China. In that circumstances, the number of patients was large and medical resources were limited. Due to the higher transmissibility and high mortality of COVID-19, only partially patients' electrocardiogram assessment was performed. All COVID-19 patients' coronary angiography was not conducted. Therefore, we can't ascertain whether the elevation of troponin is due to myocarditis or to acute coronary syndromes. Moreover, we also cannot exclude that patients died for acute coronary syndromes or because of heart failure after a massive myocarditis. Thirdly, SARS-CoV-2 injection not only evokes inflammation storm, but also damages myocardial cells directly. Elevation of inflammatory cytokines also damage myocardial cells. So, the present study can't ascertain the exact mechanism of SARS-CoV-2 inducing myocardial injury.

## Conclusion

In summary, the present study mainly analyzed SARS-CoV-2-evoked myocardial injury among COVID-19 patients in two hospitals from different region. These results showed that SARS-CoV-2-induced myocardial injury was more general in critically ill patients. Furthermore, male elderly COVID-19 patients with hypertension were more vulnerable to myocardial injury. Our results firstly suggest that myocardial injury at early stage elevates the risk of death of COVID-19 patients. What's more, SARS-CoV-2-evoked myocardial injury has not completely recovered in 14 days after discharge. Therefore, it is essential to further investigate whether SARS-CoV-2 results in a long-term myocardial injury.

## Data Availability Statement

All datasets generated for this study are included in the article/Supplementary Material.

## Ethics Statement

All procedures were performed in accordance with the ethical standards of the responsible committee on human experimentation (institutional and national) and with the Helsinki Declaration of 1975. This study was approved by the institutional ethics board of Union Hospital of Huazhong University of Science and Technology, and Second People's Hospital of Fuyang City. All COVID-19 patients were eligible in this study. Oral consent was obtained from patients or patients' next of kin.

## Author Contributions

D-XX and HZ designed the research. LF, X-YL, JF, H-XX, YX, M-DL, F-FL, and YL conducted the research. LF, X-YL, and JF analyzed the data. D-XX and LF wrote the paper and had primary responsibility for final content. All authors read and approved the final manuscript.

## Conflict of Interest

The authors declare that the research was conducted in the absence of any commercial or financial relationships that could be construed as a potential conflict of interest.
