# Supplementary material for: Myocardial Injury at Early Stage and Its Association With the Risk of Death in COVID-19 Patients: A Hospital-Based Retrospective Cohort Study
Source: Front Cardiovasc Med. 2020 Oct 30;7:590688. doi: 10.3389/fcvm.2020.590688 (PMC7661636; doi:10.3389/fcvm.2020.590688)
Supplement: Supplementary file 1 [file Table_1.docx]

Supplemental Table 1. The associations of myocardial injury indexes with oxygenation index and CRP.

| Parameter | Statistic | Myocardial injury indexes | | | |  |
| --- | --- | --- | --- | --- | --- | --- |
|  |  | AST | LDH | CKMB | CK | Tn |
| Oxygenation index | *r* | -0.249 | -0.431 | -0.081 | -0.115 | -0.221 |
|  | *P* | 0.001 | ＜0.001 | 0.291 | 0.119 | 0.038 |
| CRP | *r* | 0.241 | 0.457 | -0.027 | -0.198 | 0.135 |
|  | *P* | 0.004 | ＜0.001 | 0.747 | 0.018 | 0.121 |
